# Upconversion Luminescence of Silica–Calcia Nanoparticles Co-doped with Tm^3+^ and Yb^3+^ Ions

**DOI:** 10.3390/ma14040937

**Published:** 2021-02-16

**Authors:** Katarzyna Halubek-Gluchowska, Damian Szymański, Thi Ngoc Lam Tran, Maurizio Ferrari, Anna Lukowiak

**Affiliations:** 1 Institute of Low Temperature and Structure Research, Polish Academy of Sciences, ul. Okolna 2, 50-422 Wroclaw, Poland; d.szymanski@intibs.pl; 2 IFN-CNR CSMFO Lab. and FBK Photonics Unit, Via alla Cascata 56/C, Povo, 38100 Trento, Italy; thitran@fbk.eu (T.N.L.T.); maurizio.ferrari@ifn.cnr.it (M.F.)

**Keywords:** bioactive glass, upconversion, ytterbium, thulium

## Abstract

Looking for upconverting biocompatible nanoparticles, we have prepared by the sol–gel method, silica–calcia glass nanopowders doped with different concentration of Tm^3+^ and Yb^3+^ ions (Tm^3+^ from 0.15 mol% up to 0.5 mol% and Yb^3+^ from 1 mol% up to 4 mol%) and characterized their structure, morphology, and optical properties. X-ray diffraction patterns indicated an amorphous phase of the silica-based glass with partial crystallization of samples with a higher content of lanthanides ions. Transmission electron microscopy images showed that the average size of particles decreased with increasing lanthanides content. The upconversion (UC) emission spectra and fluorescence lifetimes were registered under near infrared excitation (980 nm) at room temperature to study the energy transfer between Yb^3+^ and Tm^3+^ at various active ions concentrations. Characteristic emission bands of Tm^3+^ ions in the range of 350 nm to 850 nm were observed. To understand the mechanism of Yb^3+^–Tm^3+^ UC energy transfer in the SiO_2_–CaO powders, the kinetics of luminescence decays were studied.

## 1. Introduction

Rare-earth-activated systems have been demonstrated to be the pillar of photonic technologies enabling a broad spectrum of crucial applications in strategic social and economic priorities [1,2,3,4,5]. Systems based on rare-earth-doped upconverters are largely employed in bioimaging, drug delivery, laser, lighting, photon management, environmental sensing, and nanothermometry [6,7,8,9,10,11,12,13,14,15]. Upconversion (UC) mechanism is a process of energy transfer from a sensitizer in a proper host matrix, which is excited under low-energy radiation (usually near infrared, NIR), to an emitter that emits higher energy photons than the excitation ones. This is a multi-photon process in which two or more low-energy photons are needed to generate in consequence shorter wavelength photon and the emission with higher photon energy (ultraviolet (UV), visible (VIS), or NIR radiation) than the energy of the pumping source [16,17].

Yb^3+^ ions are excellent luminescence sensitizers for other rare earth ions due to their effective absorption cross section at 980 nm [16]. The Yb^3+^ ion has been demonstrated to be a crucial element for upconverters [16], high power lasers [18], photovoltaic systems [19], and integrated optics [5]. The couple Tm^3+^/Yb^3+^ ions have been largely investigated because of the need to efficiently pump the Tm^3+^ ion to obtain upconversion-based photonic devices on a broad spectrum of wavelengths. There are reports on Tm^3+^/Yb^3+^ upconversion luminescence in single crystals [9], nanocrystals [17], glasses [20], ceramics [21], and glass-ceramics [19]. Interesting research was performed on Tm^3+^/Yb^3+^ upconversion luminescence in silicate glasses [22,23] to enhance the optical amplification spectroscopic properties of thulium in silica. Moreover, studies were also dedicated to the downconversion mechanism between Tm^3+^/Yb^3+^ in glass or nanocrystals [17,24].

Properly synthesized silica-based glasses can be described as bioactive materials able to form a hydroxyapatite-like surface layer when immersed in a simulated body fluid similar to the blood plasma. Non-toxic and biocompatible bioactive glass is used in medicine to replace defects in bones or to stimulate growing new bones. Recently, lanthanides co-doped with ytterbium ions were introduced into bioactive glass matrices to study their optical properties and upconversion luminescence. For example, Li et al. described a couple of Er^3+^/Yb^3+^ ions in CaSiO_3_ [25] and Kalaivani et al. doped sol–gel-derived glasses of SiO_2_–Na_2_O–CaO–P_2_O_5_ with Tb^3+^ and Yb^3+^ [26]. The systems were tested to estimate their biological properties and applications for regenerative medicine. In addition to conducting a bioactivity test of Gd^3+^/Yb^3+^-co-doped SiO_2_–Na_2_O–CaO–P_2_O_5_ glass [27], the group of Borges had also a second goal to study the role of rare earth ions in the modification of glass structure [28]. 

Within this work, we successfully used the sol–gel method to prepare powders of silica–calcia glass doped with different concentrations of Tm^3+^ and Yb^3+^ ions (not studied so far) and characterized their structure and morphology. The spectroscopic properties of the samples, such as absorption, UC emission, and photoluminescence decay times, were also investigated to propose the energy transfer mechanism in the studied system.

## 2. Materials and Methods

### 2.1. Synthesis of SiO_2_–CaO Particles Doped with Rare Earth Ions

The analytical reagents comprising tetraethyl orthosilicate (TEOS, Sigma-Aldrich, Darmstadt, Germany), HNO_3_, NH_4_OH, Ca(NO_3_)_2_∙4H_2_O (Avantor Performance Materials, Gliwice, Poland), and high purity Yb_2_O_3_ and Tm_2_O_3_ (≥99.99%, Stanford Materials Co., Lake Forest, CA, USA) were used as starting materials to obtain seven samples of different compositions varied through the incorporation of rare earth elements: (xTm^3+^/yYb^3+^):63SiO_2_–37CaO, where x = 0.15, 0.3 or 0.5 and y = 0, 1, 2, 3 or 4 (mol%). Additionally, one sample without rare earth ions was synthesized as well. 

The silica–calcia glasses were prepared by the sol–gel method as described previously [29]. The starting materials were mixed in requisite proportions and order. The derived powders were centrifuged and washed three times with distilled water and dried at 70 °C overnight. Then, the samples were calcined at 800 °C for 2 h.

### 2.2. Characterization

The structure of the samples was identified by X-ray diffractometer (XRD, X’Pert PRO, PANalytical, Bruker, Germany) with Cu Kα radiation (λ = 1.5406 Å) scanning the diffraction angles (2θ) between 10° and 70° at room temperature. 

The morphology and microstructure were examined by a Transmission Electron Microscope (TEM) (Philips CM-20 SuperTwin, Eindhoven, The Netherlands, operating at 160 kV) equipped with a selected area electron diffraction (SAED). TEM measurements have been performed on a copper grid coated with carbon. The grain size distribution has been determined for about 200 particles. The chemical composition of the samples was determined using a Field Emission Scanning Electron Microscope (FE-SEM) (FEI Nova NanoSEM 230, Fremont, CA, USA) equipped with an energy dispersive X-ray spectrometer (EDAX Genesis XM4). The EDS analyses were performed at 18.0 kV from the large area (250 μm × 200 μm) of the samples. The powder samples were included in the carbon resin and then pressed to obtain a large and flat area. Signals from three randomly selected areas were collected to ensure satisfactory statistical averaging.

The absorption spectra of the powders were collected in the reflection mode using a UV-VIS-NIR spectrophotometer (Agilent Cary 5000, Santa Clara, CA, USA) in the wavelength range from 200 to 1300 nm. Photoluminescence spectra were measured using an FLS980 fluorescence spectrometer (Edinburgh Instruments, Livingstone, UK) equipped with a 980 nm laser diode as an excitation source and a photomultiplier as a detector. For decay time measurements, triggering was used with a 150 W xenon pulsed flash lamp (pulse duration 1–2 µs, 20 Hz) and the same photomultiplier as a detector. Spectra were corrected with respect to the detector sensitivity and lamp characteristic. All the measurements were done at room temperature. From the measured photoluminescence decay curves, 1/e decay times (τ_1/e_) were determined for all samples [30].

## 3. Results and Discussion

### 3.1. Structural and Morphological Characterization

**Figure 1:**
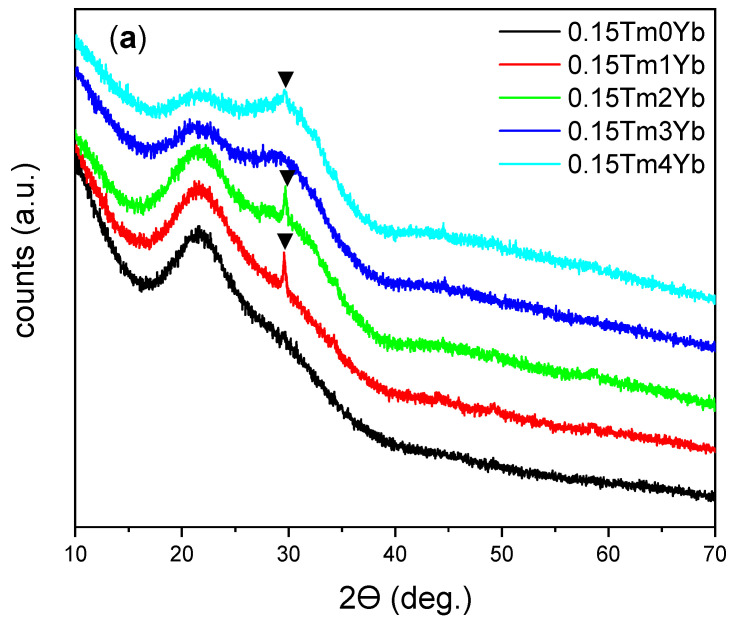

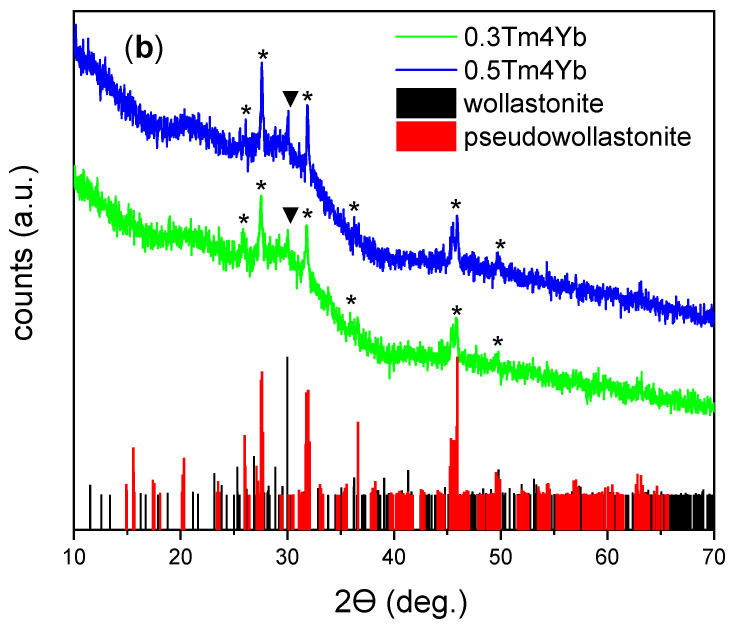
XRD patterns of the samples with various rare earth ions concentrations: (**a**) different Yb^3+^ concentrations (0, 1, 2, 3, and 4 mol%) and the same Tm^3+^ concentration (0.15 mol%); (**b**) different Tm^3+^ (0.3 and 0.5 mol%) and the same Yb^3+^ concentration (4 mol%) (▼—wollastonite and ⁎—pseudowollastonite phase of CaSiO_3_ assigned to JCPDS Nos. 96-900-5779 and 96-900-2180 patterns, respectively).

The chosen method allowed the preparation of two series of samples with various Tm^3+^ and Yb^3+^ concentrations (Table 1). The XRD patterns of all compositions are shown in Figure 1. All patterns were characterized by signals displaying the amorphous nature of the glass with typical broad reflections. For three samples with the lowest thulium concentration of 0.15% (Figure 1a), a low reflection signal at 2θ = 30° was recorded indicating partial crystallization of the glass probably in the wollastonite phase of calcium silicate (CaSiO_3_, JCPDS No. 96-900-5779). Due to the low samples’ crystallinity, this phase cannot be fully confirmed but it is pointed out here to be the most probable. In the case of samples doped with 4% of Yb^3+^ and a higher concentration of Tm^3+^ ions (Figure 1b), a higher degree of crystallization occurred and the second phase of calcium silicate was detected—pseudowollastonite (JCPDS No. 96-900-2180). Thus, it can be concluded that the introduction of selected rare earth ions in the system favors the formation of glass-ceramic.

**Figure 2:**
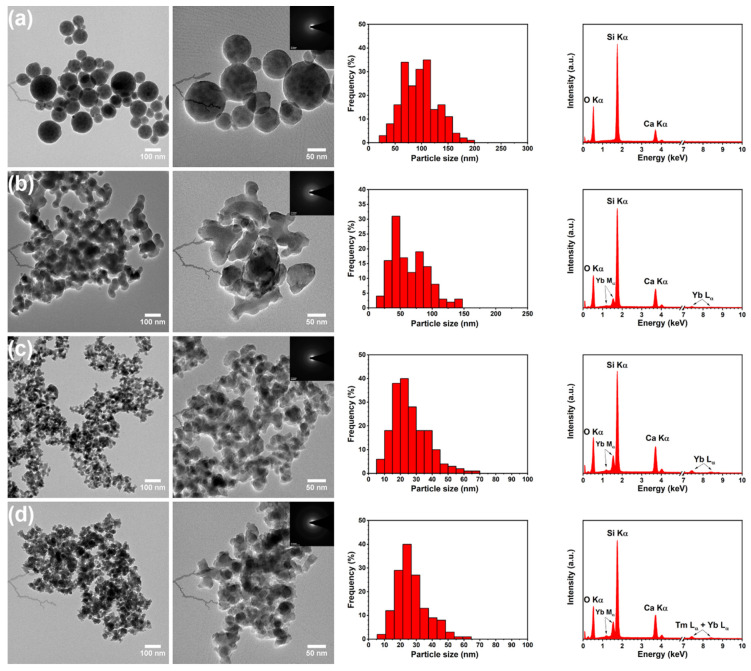
TEM images (on the left) with selected area electron diffraction (SAED) (as insets), histograms of size particle distribution (in the middle), and EDS spectra (on the right) of (**a**) un-doped (SiO_2_-CaO), (**b**) 0.15Tm2Yb, (**c**) 0.3Tm4Yb, and (**d**) 0.5Tm4Yb samples.

In order to determine the morphology of the silica–calcia powders, a transmission electron microscope was used. Figure 2 shows representative TEM images of the un-doped and Tm^3+^/Yb^3+^-co-doped samples, which reveal that examined SiO_2_–CaO particles were nanosized and exhibited two different morphologies. TEM image of the un-doped glass and the particle size distribution (Figure 2a) indicated that SiO_2_–CaO had a regular morphology and relatively good monodispersity with diameters of particles from 80 to 120 nm. It can also be seen that spherical particles were non-aggregated and uniformly distributed. On the other hand, the particles of Tm^3+^/Yb^3+^-doped samples exhibited undefined shapes and formed large groups of agglomerates (see Figure 2b–d). The size distribution histograms showed that the maximum size of particles decreased from 30–80 nm to 15–30 nm for the 0.15Tm2Yb and 0.5Tm4Yb samples, respectively. As is well known, the sol–gel synthesis conditions strongly influence the final morphology of the samples. This effect is easily observed in the case of synthesis of silica–calcia system where both particles of undefined shape as well as spheres with different diameters can be obtained [29]. From these studies, it is evident that even a low concentration of introduced rare earth ions modifies synthesis conditions and effects in different shapes of the particles.

To confirm the structure of SiO_2_–CaO samples, SAED images were also employed. The selected area electron diffraction pattern displayed an amorphous pattern of SiO_2_–CaO glass (see insets in Figure 2). It is clearly visible that a diffraction pattern of examined samples exhibited a ring pattern as a result of the absence of long-range order, which is consistent with the XRD results showing broad reflections from the amorphous phase. SAED technique has not shown the minor crystalline phases, most probably due to the analysis performed on a selected area of the samples.

The elemental compositions of the SiO_2_–CaO glasses were analyzed by Energy Dispersive X-ray Spectroscopy. The EDS spectra presented in Figure 2 show three strong bands of oxygen (O), silicon (Si), and calcium (Ca). Other recorded bands with maxima at 7.15 and 7.42 keV have been attributed to thulium (Tm) and ytterbium (Yb) elements, respectively. Moreover, additional bands from other elements were not detected which indicates that the samples were free from contaminants. Table 1 shows the weight percentage of the SiO_2_, CaO, Tm_2_O_3_, and Yb_2_O_3_ obtained from the EDS analysis.

### 3.2. Spectroscopic Properties

#### 3.2.1. Absorption Spectra

**Figure 3:**
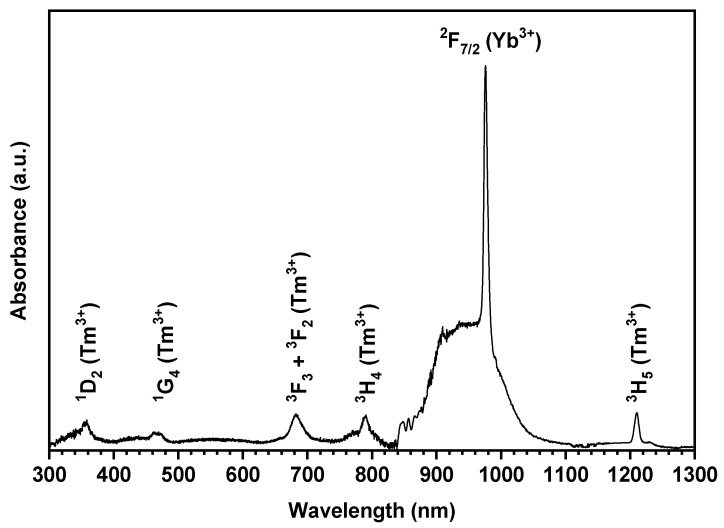
Absorption spectra of the 0.5Tm4Yb sample.

The absorption spectrum of 0.5Tm4Yb powder in the NIR-Vis spectral range is shown in Figure 3. In the 300–1300 nm range, there were observed relatively intense and well separated bands of intraconfigurational *4f^N^* → *4f^N^* transitions from ^3^H_6_ and ^2^F_5/2_ ground state to the high-lying levels of Tm^3+^ and Yb^3+^ ions, respectively. The absorption bands with maxima at 356, 463, 683, and 1210 nm have been assigned to the transitions from the ^3^H_6_ ground state to the ^3^H_5_, ^3^H_4_, <^3^F_3_, ^3^F_2_>, ^1^G_4_, and ^1^D_2_ excited states of Tm^3+^, respectively. The absorption bands related to the higher energy level of trivalent thulium ion were not observed due to the strong absorption of the SiO_2_–CaO host and scattering of the light on the sample.

The absorption band located at 840–1135 nm, assigned to the spin-allowed ^2^F_7/2_ → ^2^F_5/2_ transition of Yb^3+^ (present in the sample at a higher concentration than Tm^3+^ ions), exhibits the maximum value of absorbance. The observed shape of the Yb^3+^ absorption band showed inhomogeneous broadening features due to the short-range order in the amorphous matrix. This band consisted of broad weakly resolved bands located between 840 and 1110 nm, which corresponded to the Stark components of the ^2^F_7/2_ → ^2^F_5/2_ transitions. The strongest absorption line is the 0-phonon line at around 976 nm. This is the transition from the lowest Stark level of the ^2^F_7/2_(1) ground state to the lowest Stark level of the ^2^F_5/2_(5) excited state. The others, much less intensive and less separated components, are more difficult to spectrally resolve. 

#### 3.2.2. Emission Spectra

**Figure 4:**
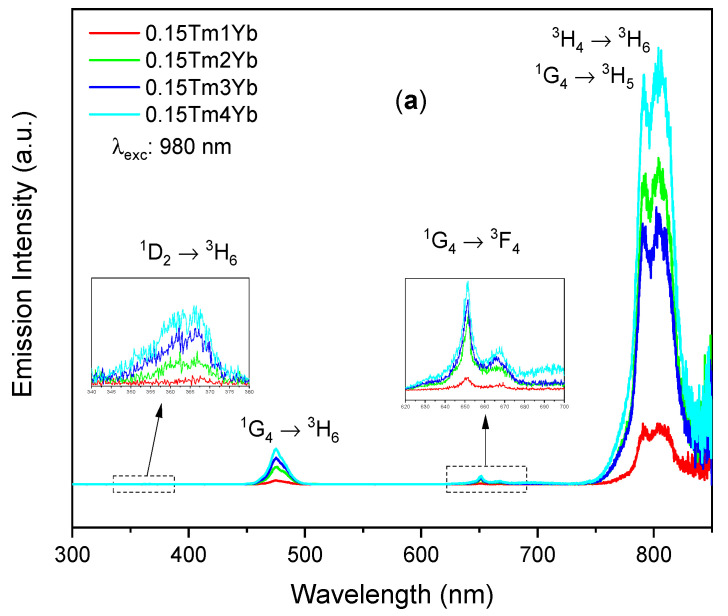

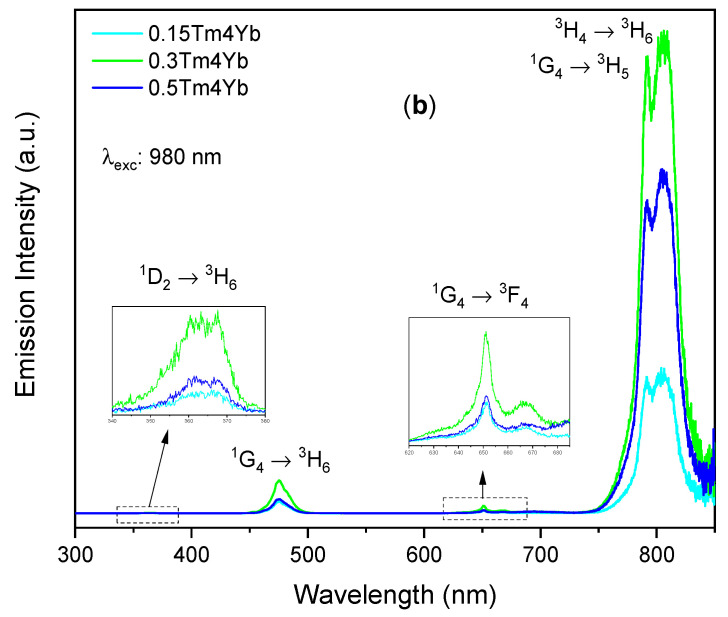
Upconversion emission spectra of silica–calcia matrix doped with different (**a**) Yb^3+^ (1, 2, 3, and 4 mol%) and (**b**) Tm^3+^ (0.15, 0.3, and 0.5 mol%) concentrations.

The upconversion emission spectra shown in Figure 4 were registered under near infrared excitation (980 nm) to study the energy transfer between Yb^3+^ and Tm^3+^ at various active ions concentrations. In these spectra, four emission bands in the ultraviolet (centered at 366 nm), blue (475 nm), red (651 nm), and near infrared (805 nm) were observed and all the emission bands corresponded to the Tm^3+^ intraconfigurational *f-f* transitions. The ultraviolet emission band is assigned to the ^1^D_2_ → ^3^H_6_ transition while the blue and the red emission bands are corresponding to the ^1^G_4_ → ^3^H_6_ and the ^1^G_4_ → ^3^F_4_ transitions, respectively. In particular, the NIR band can be contributed by two different transitions: ^1^G_4_ → ^3^H_5_ and ^3^H_4_ → ^3^H_6_, of which the luminescence bands overlap at around 800 nm region as reported in the previous works [23,31,32]. From both sets of spectra shown in Figure 4, one can see that the most intense upconversion emission was the NIR emission. At the same constant concentration of Tm^3+^ (0.15%), increasing Yb^3+^ concentration up to 4% led to an enhancement of all the observed UC emission bands (Figure 4a). Most probably, this effect arose due to a more efficient energy transfer between Yb^3+^ and Tm^3+^ ions at a higher concentration of the donor. At the same, Yb^3+^ concentration of 4%, the UC emission bands were enhanced when the Tm^3+^ concentration increased from 0.15% to 0.3%, but they diminished when the Tm^3+^ concentration reached 0.5% (Figure 4b). A higher concentration of acceptor (Tm^3+^) might also favor energy migration observed as more intense luminescence, but above some level of Tm^3+^, concentration quenching may occur. 

#### 3.2.3. Time-Resolved UC Photoluminescence

In this section, the time-resolved UC photoluminescence in the visible and NIR ranges are analyzed separately. The emission intensity in the UV, i.e., at 366 nm, had a very low intensity and therefore, these bands were not taken into consideration in the studies. 

**Figure 5:**
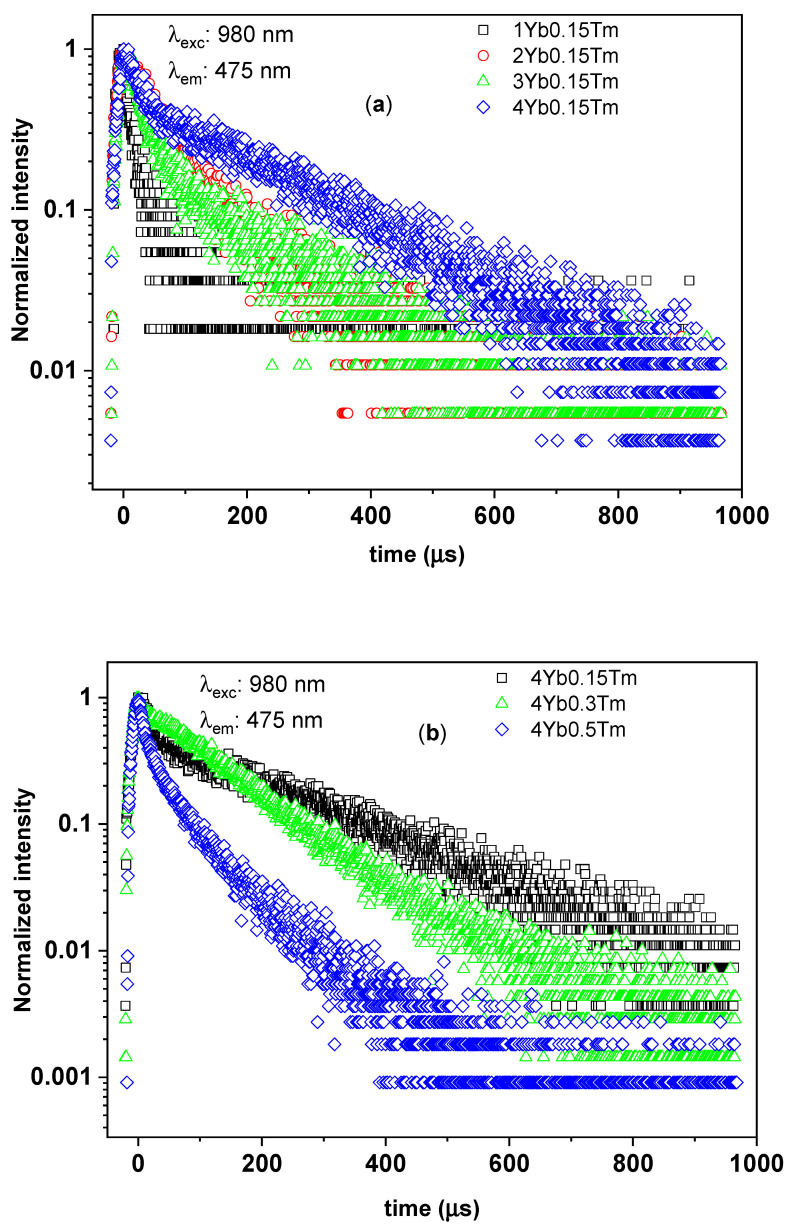
The time-resolved upconversion (UC) photoluminescence of the branching of ^1^G_4_–^3^H_6_ transition from ^1^G_4_ manifolds of Tm^3+^ in silica–calcia matrix doped with: (**a**) different Yb^3+^ concentrations (1, 2, 3, and 4 mol%) and the same Tm^3+^ concentration (0.15 mol%); (**b**) different Tm^3+^ concentrations (0.15, 0.3, and 0.5 mol%) and the same Yb^3+^ concentration (4 mol%).

**Figure 6:**
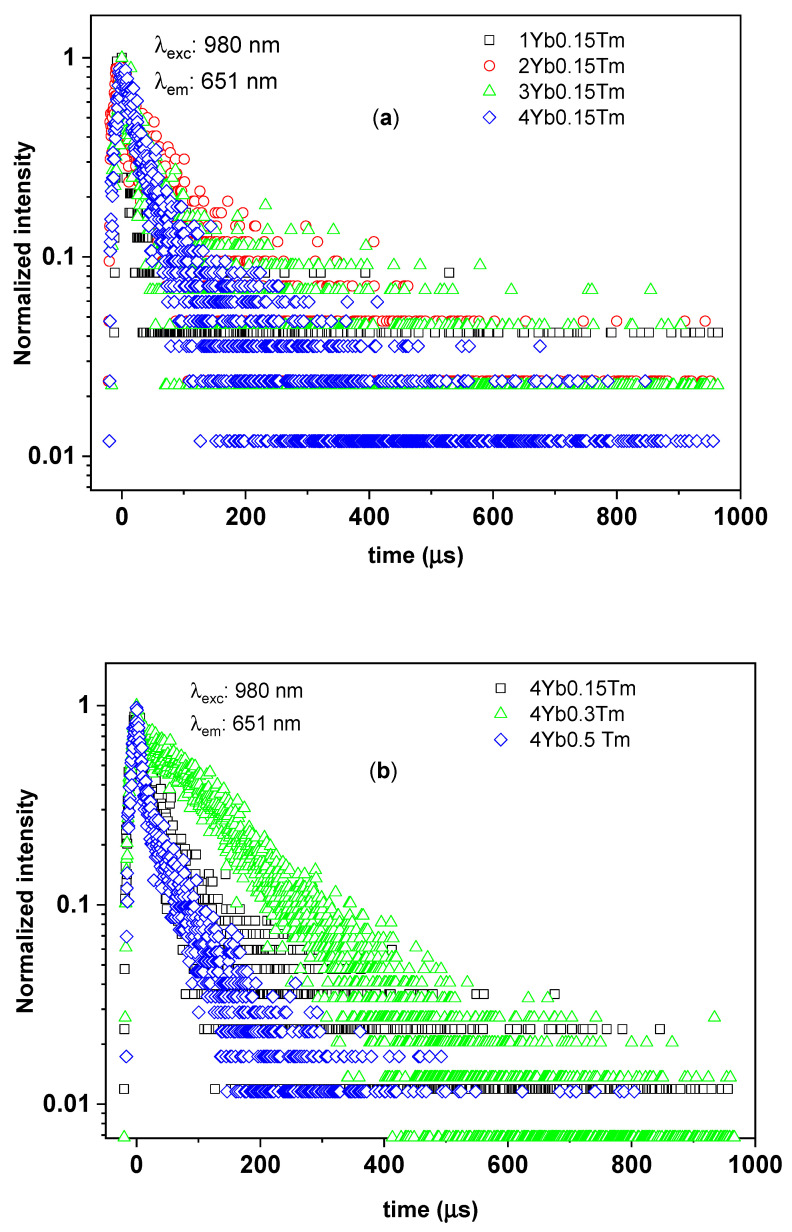
The time-resolved UC photoluminescence of the branching of ^1^G_4_–^3^F_4_ transition from ^1^G_4_ manifolds of Tm^3+^ in silica–calcia matrix doped with: (**a**) different Yb^3+^ concentrations (1, 2, 3, and 4 mol%) and the same Tm^3+^ concentration (0.15 mol%); (**b**) different Tm^3+^ concentrations (0.15, 0.3, and 0.5 mol%) and the same Yb^3+^ concentration (4 mol%).

To study the luminescence decay times of the ^1^G_4_ manifolds of Tm^3+^ in silica–calcia co-doped with different Tm^3+^ and Yb^3+^ concentrations, the time-resolved UC photoluminescence spectra of the branching ^1^G_4_ → ^3^H_6_ transition (at 475 nm) and ^1^G_4_ → ^3^F_4_ transition (at 650 nm) were measured as shown in Figure 5 and Figure 6, respectively. In the particular case of the SiO_2_–CaO powder co-doped with 0.3% Tm^3+^ and 4% Yb^3+^ (0.3Tm4Yb), the time-resolved luminescence spectra of both branching ^1^G_4_ → ^3^H_6_ and ^1^G_4_ → ^3^F_4_ transitions showed a single exponential decay with comparable values of ^1^G_4_ decay times of around 100 µs (see Table 2). This single exponential decay (indicating that only one site of Tm^3+^ ions in the 0.3Tm4Yb powder is involved in these UC processes) is in nature with the characteristic lifetime of the ^1^G_4_ manifolds of Tm^3+^ reported in cases of Tm^3+^-single-doped silica fibers [23,33,34] and thus, it is suggested to be the spontaneous decay time of the ^1^G_4_ manifolds of Tm^3+^ in the SiO_2_–CaO matrix. In comparison with the ^1^G_4_ decay times of Tm^3+^ in fluorides being typically in ms range, these ions in silica-based matrices exhibit a shorter luminescence lifetime [35] indicating a strong contribution from non-radiative pathways. This was expected due to the chemical composition of the network where [SiO_4_]^4−^ groups give rise to the vibrational energies in the order of 1100 cm^−1^ (relatively high compared to other host materials, such as fluorides (350 cm^−1^) or tellurites (700 cm^−1^)). Additionally, possible defective structure on a microscopic level of the sol–gel-derived SiO_2_–CaO glassceramic can also induce the shortening of the decay times of Tm^3+^.

The powders other than 0.3Tm4Yb exhibited non-single exponential decays for both branching ^1^G_4_ → ^3^H_6_ and ^1^G_4_ → ^3^F_4_ transitions of Tm^3+^ with shorter 1/e decay times. As discussed in Section 3.1, the structure of the SiO_2_–CaO changed with the dopants concentrations. These changes most probably give rise to the presence of multiple, crystallographically inequivalent sites due to the short-range order in the glassceramics [2]. Consequently, this can induce the complex features and shortening of the decay times in the investigated samples. Moreover, the shortening of the 1/e decay times of the ^1^G_4_ manifolds can be contributed as well by several processes such as cross-relaxation between Tm^3+^ ions and back energy transfer from Tm^3+^ to Yb^3+^ [23,36]. In other words, there are several decay processes possibly occurring at the depletion of the ^1^G_4_ populated ions but further investigations would be needed to describe them in detail.

To investigate more deeply the effect of Tm^3+^ and Yb^3+^ concentrations on the UC photoluminescence, the 1/e decay times of the ^1^G_4_ manifolds for all the samples were collected in Table 2 and analyzed. The decay times of ^1^G_4_ manifolds derived from both branching transitions ^1^G_4_ → ^3^H_6_ and ^1^G_4_ → ^3^F_4_ of the investigated powders showed the same behavior concerning the concentration of the dopants, as well as their Yb^3+^:Tm^3+^ concentration ratios varying from 7 to 27 (Table 2). In the SiO_2_–CaO samples doped with the same Tm^3+^ concentration of 0.15%, the powders doped with the smallest Yb^3+^ concentration, i.e., 1%, with Yb^3+^:Tm^3+^ ratio equal to 7, exhibited a short 1/e lifetime of the ^1^G_4_ manifolds in the range of 13–15 µs. The 0.5Tm4Yb sample having almost the same Yb^3+^:Tm^3+^ ions ratio (equal to 8) showed a similar 1/e lifetime. Considering the powders doped with 0.15% of Tm^3+^ and higher Yb^3+^ concentrations (2, 3, and 4%), the 1/e decay times showed comparable longer values in the range from 23 to 63 µs. On the other hand, concerning the SiO_2_–CaO powders doped with a constant Yb^3+^ concentration of 4%, they have not shown a clear tendency of the variation of the 1/e decay times with Tm^3+^ content. Among them, the 0.3Tm4Yb sample exhibited the longest ^1^G_4_ decay time of about 100 µs.

The above observations may rise to the following conclusions. The 0.3Tm4Yb powder showed a single exponential ^1^G_4_ decay times indicating that the processes of spontaneous decays from the ^1^G_4_ manifolds to other lower energy electronic states of the Tm^3+^ ions dominated the depletion of such ions at the excited ^1^G_4_ state. Moreover, the possible impact of the multiple, crystallographically inequivalent sites, back energy transfer from Tm^3+^ to Yb^3+^, and non-radiative processes were negligible in the de-population of Tm^3+^ ions at ^1^G_4_ manifolds for this sample. For other Yb^3+^:Tm^3+^ concentration ratios, especially at low Yb^3+^:Tm^3+^ ratios of 7 or 8, fast decay processes may take place evidenced by the non-single-exponential behaviors and shortening of the ^1^G_4_ decay times (see Figure 5 and Figure 6). The aforementioned processes absent in the case of 0.3Tm4Yb may derive the depletion of the ^1^G_4_ populated ions in the other samples. Eventually, the SiO_2_–CaO powder co-doped with 0.3% Tm^3+^ and 4% Yb^3+^ with their dominant spontaneous emission is the most optimal composition for obtaining upconversion photoluminescence in the blue and red spectral ranges.

**Figure 7:**
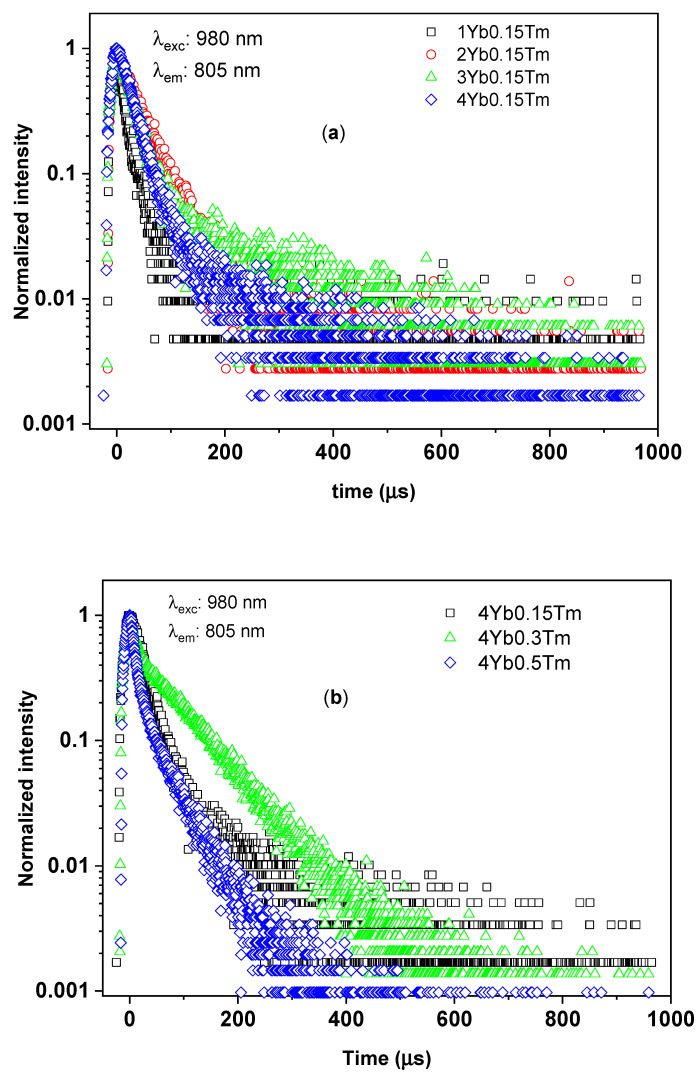
The time-resolved UC photoluminescence at 805 nm emission band of Tm^3+^ in silica–calcia matrix doped with: (**a**) different Yb^3+^ concentrations (1, 2, 3, and 4 mol%) and the same Tm^3+^ concentration (0.15 mol%); (**b**) different Tm^3+^ concentrations (0.15, 0.3, and 0.5 mol%) and the same Yb^3+^ concentration (4 mol%).

Looking at the time-resolved UC photoluminescence at 805 nm emission band of the investigated samples (Figure 7), one may note that the decay curves of the ^3^H_4_ manifolds of Tm^3+^ in all the samples exhibited non-single-exponential decays profiles. The 1/e decay time τ_1/e_ of the ^3^H_4_ manifolds of Tm^3+^ are also tabulated in Table 2. For powders with a Yb^3+^:Tm^3+^ molar ratio ranging from 13 to 27, the decay times had comparative values ranging from 31 µs to 39 µs. On the other hand, for the SiO_2_–CaO powders with a Yb^3+^:Tm^3+^ molar ratio of 7 and 8, the same parameter exhibited shorter time of about 13 µs. Thus, the 1/e decay times of the ^3^H_4_ of Tm^3+^ ions concerning dopants concentrations showed similar behavior with the 1/e ^1^G_4_ decay times. The 0.3Tm4Yb sample also exhibited the longest 1/e decay time of the ^3^H_4_ manifolds as in the case of the ^1^G_4_. However, unlike the cases of the blue and red emission bands from the ^1^G_4_ manifold, the time-resolved photoluminescence NIR band of Tm^3+^ ions of this sample did not show single-exponential behavior. These observations are not surprising since, at the 805 nm emission band, there exists an overlap of the two transitions: ^3^H_4_ → ^3^H_6_ and ^1^G_4_ → ^3^H_5_.

**Figure 8:**
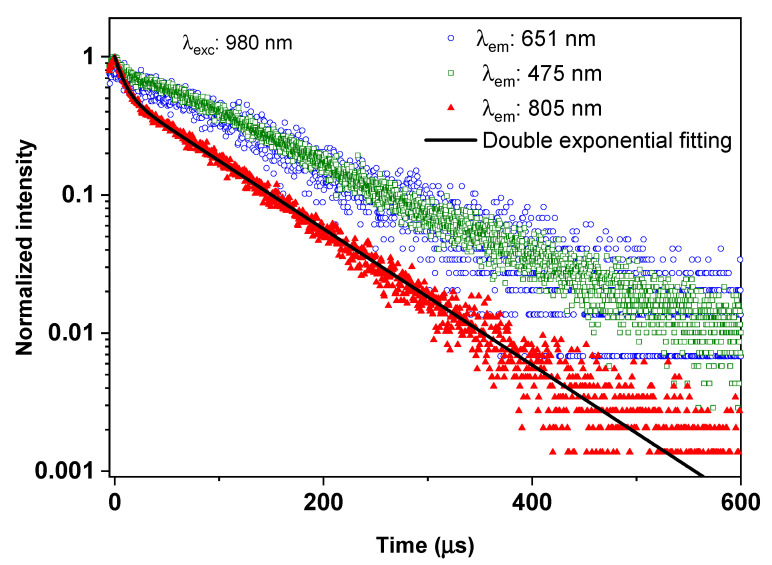
The time-resolved UC photoluminescence at 805 nm emission band of Tm^3+^ of the 0.3Tm4Yb sample and its fitting based on a double exponential function. The time-resolved UC photoluminescence at the 651 nm and 475 nm emission bands from the ^1^G_4_ manifolds are also recalled for a comparison.

To define the decay time of the ^3^H_4_ manifolds of Tm^3+^ ions as well as reveal the contribution of both ^3^H_4_ → ^3^H_6_ and ^1^G_4_ → ^3^H_5_ transitions to the emission band centered at 805 nm, the time-resolved UC photoluminescence at 805 nm emission band of the 0.3Tm4Yb sample was fitted into a double exponential function as shown in Figure 8 together with the decay curves from ^1^G_4_ manifolds, i.e., blue and red emission band. The fitting result is listed in Table 3. 

As one can see from Figure 8, the time-resolved UC photoluminescence at 805 nm emission band of Tm^3+^ of the 0.3Tm4Yb powder is well-fitted to the double exponential function. Thus, a sum function of two exponentials was employed to describe the luminescence decay function as suggested by Righini et al. [30]:

(1) $$\phi \left(t\right)=A\times \exp\left[-\frac{t}{\tau_{1}}\right]+\left(1-A\right)\exp\left[-\frac{t}{\tau_{2}}\right]$$

where ϕ(t) is the decay function, τ_1_ and τ_2_ are the two decay time components, and A is the fitting parameter describing the amplitude of the contribution of the decay time component τ_1_ (Table 3).

The double exponential fitting results unravel the contribution of both the ^1^G_4_ → ^3^H_5_ and ^3^H_4_ → ^3^H_6_ transitions. The short component of around 9 µs is assigned to the decay time of the ^3^H_4_ manifolds, which is slightly lower than the value of around 20 µs reported in the literature [23]. This reduction of the ^3^H_4_ manifold decay time can be due to the multiphonon processes or cross-relaxation in the SiO_2_–CaO matrix. The long decay component of around 88 µs which is comparable to the ^1^G_4_ decay time (see Table 3) indicates the contribution of the ^1^G_4_ → ^3^H_5_ transition to the UC photoluminescence at 805 nm. Moreover, the obtained fitting parameter A of 0.55 indicates that both ^1^G_4_ → ^3^H_5_ and ^3^H_4_ → ^3^H_6_ transitions contributed comparably to the UC photoluminescence at 805 nm.

It must be also mentioned that the existence of the crystalline phases in selected samples may influence the kinetics of the photoluminescence processes because the decay times of thulium ions depend also on the matrix [37,38].

#### 3.2.4. UC Excitation Power Dependence and Yb^3+^-Tm^3+^ UC Energy Transfer

**Figure 9:**
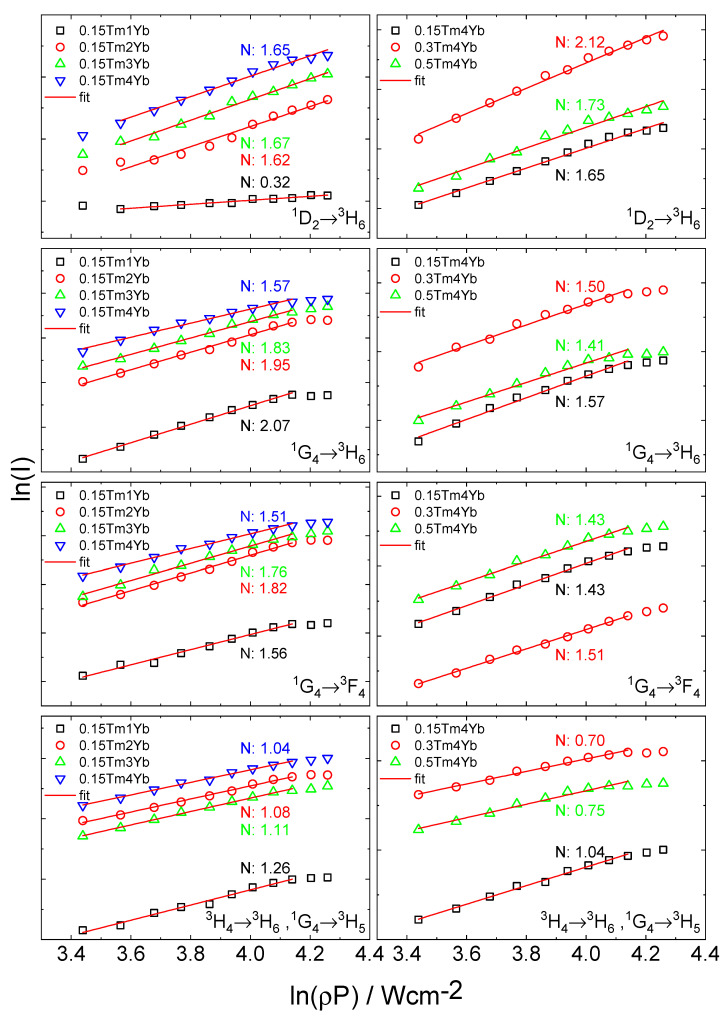
A log-log plot of the dependence of luminescence intensities (bands at 366 nm, 475 nm, 651 nm, and 805 nm) on the laser power density of excitation source for samples co-doped with different concentration of Tm^3+^ and Yb^3+^ ions.

The upconversion emission intensity for the observed UV, blue, red, and NIR signals was examined as a function of the laser diode (980 nm) excitation power density (Figure 9). 

In general, the number of photons which are required to populate the upper emitting state under unsaturated conditions can be obtained by the relation [39,40]:

I_L_ ∝ P^N^ (2)

where I_L_ is the photoluminescence intensity, P is the pump laser power density (W·cm^−2^), and N is the number of the laser photons required to observe UC emission. At low incident power at which the unsaturated condition is satisfied, the UC photoluminescence intensity is proportional to the incident pump power following the relation (2). Therefore, the slope values N in Figure 9 can provide information on the number of photons absorbed to provide upconversion photoluminescence. The values are determined from the linear dependencies range as marked in Figure 9 (points at high incident powers are not taken into account due to the saturation effect [41]). The summary of N numbers for different UC processes observed as emission at 366 nm, 475 nm, 651 nm, and 805 nm for the investigated samples is listed in Table 4. 

The slope value N of all transitions varies slightly upon the concentrations of the active ions in the investigated samples. It was concluded that in the ^3^H_4_ → ^3^H_6_ transitions, absorption of one photon is required whereas, for other transitions, two or more photons are involved in the upconversion process. The unexpected low value of N = 0.32 for ^1^D_2_ → ^3^H_6_ transition in the case of 0.15Tm1Yb sample was not taken into account because of the difficulties to correctly estimate the power dependence due to the low intensity of this luminescence.

**Figure 10:**
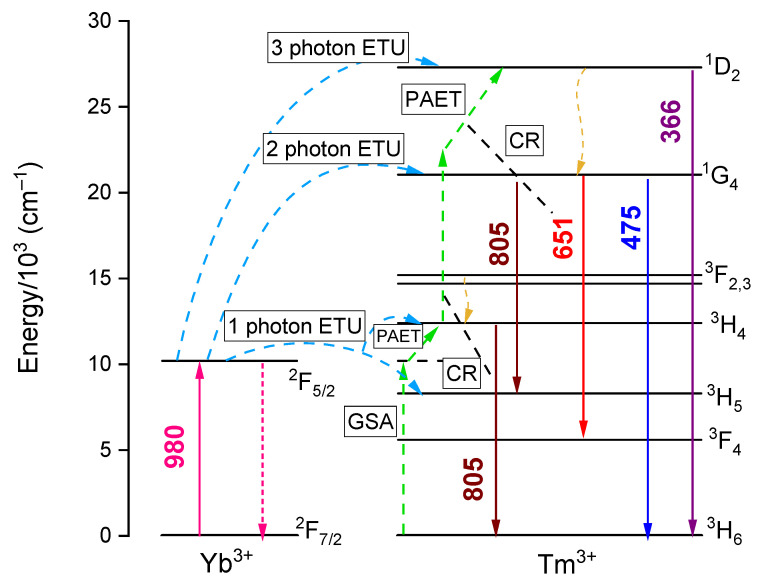
Simplified energy-level diagram of the Yb^3+^ and Tm^3+^ ions and the proposed UC mechanism from different energy states of Tm^3+^ ions. ETU—energy transfer upconversion; GSA—ground state absorption; PAET—phonon assistance upconversion; CR—cross-relaxation.

To understand the mechanism of Yb^3+^-Tm^3+^ UC energy transfer, the energy level diagrams of the Yb^3+^ and Tm^3+^ ions were prepared as illustrated in Figure 10. Taking into account the NIR upconversion at 805 nm, as discussed above, two main transitions are contributing to this emission band: the ^1^G_4_ → ^3^H_5_ and ^3^H_4_ → ^3^H_6_ transitions. Concerning the latter one, under 980 nm excitation, the population of ^3^H_4_ manifolds of Tm^3+^ can be derived by one photon energy transfer from Yb^3+^ ion at the excited state ^2^F_5/2_ (1 photon ETU; mentioned here possible energy transfer processes are marked in Figure 10) and the missing energy gap between ^2^F_5/2_ (Yb^3+^) and ^3^H_4_ states in this process can be fulfilled by phonons, i.e., phonon assistance upconversion (PAET). However, there is another possibility that several Tm^3+^ ions at a ground state can be excited into the ^3^H_4_ by the cross-relaxation processes (CR) between the excited Tm^3+^ ions and the Tm^3+^ ions at the ground state, i.e., ground state absorption (GSA). This can be an explanation for the complex decay times behavior of the investigated powders in the observed UC photoluminescence. With this UC mechanism, the number of 980 nm excitation photons needed is 1. On the other hand, referring to the ^1^G_4_ → ^3^H_5_ transition, the UC mechanism to populate the Tm^3+^ into the ^1^G_4_ is assigned to a two-photon energy transfer from Yb^3+^ ion at the excited state ^2^F_5/2_ to the Tm^3+^ ions and the necessary number of 980 nm excitation photon is 2 (2 photon ETU). If there is only the combination of these two UC energy transfers according to the excitation to ^1^G_4_ and ^3^H_4_ manifolds, the slope number of this transition should be in the range from 1 to 2 as in the cases of the samples with the lowest Tm^3+^ concentration (0.15%). However, since there can be the occurrence of the GSA and CR processes and contribution of phonon energy, the slope number of the powder can be lower than 1 as also observed in the work of Simpson et al. [23]. Furthermore, this effect probably occurs in the cases of the powders with Tm^3+^ concentration higher than 0.15%, which can be due to the higher contribution of the CR and GSA processes to the excitation of the ions to the ^3^H_4_ excited state occurring when there are higher Tm^3+^ concentration.

In the case of the UC photoluminescence at both the blue and red emission bands according to the ^1^G_4_ → ^3^H_6_ and ^1^G_4_ → ^3^F_4_ transitions, the observed slope values are ranging from 1.41 to 2.07 among the investigated powders, which is sufficiently in agreement with the proposed two-photon energy transfer from Yb^3+^ ion at the excited state ^2^F_5/2_ to the Tm^3+^ ions (2 photon ETU) as shown in Figure 10. As mentioned, the cases of the slope values below 2 can be due to the contribution of the CR processes and the contribution of multiphonon processes. 

Last but not least, concerning the UC photoluminescence at 366 nm, in principle, the three-photon energy transfer from Yb^3+^ ion at the excited state ^2^F_5/2_ to the Tm^3+^ ions (3 photon ETU) is proposed to be the mechanism driving this UV emission band. However, the slope values of the powders are generally close to 2 or even below 2. As discussed, the effect of cross-relaxation and multiphonon processes can account for this as in the other emission bands.

## 4. Conclusions

In the present work, we have successfully synthesized new Tm^3+^/Yb^3+^-co-doped SiO_2_–CaO powders by the sol–gel method. XRD analysis and transmission electron microscope images showed the influence of thulium and ytterbium ions on the structure and morphology of derived nanoparticles. For a smaller amount of dopants, the amorphous structure of the material was registered. With an increased amount of the dopants, a smaller size of the particles and partial crystallization of calcium silicate phases were observed. Furthermore, optical measurements were performed. Upconversion emission was registered upon 980 nm excitation at room temperature and four main emission bands were observed from 300 nm to 850 nm due to the transitions from ^1^D_2_ excitation level to ^3^H_6_ (ultraviolet), from ^1^G_4_ level to ^3^H_6_ (blue), ^3^F_4_ (red) and ^3^H_5_ (NIR), and from ^3^H_4_ level to ^3^H_6_ (NIR). The intensities of photoluminescence and decay times of Tm^3+^ were dependent on the concentration of both Yb^3+^ (changing from 1 to 4 mol%) and Tm^3+^ (changing from 0.15 to 0.5 mol%) ions. The highest emission intensity and single exponential decay time were registered for the concentration of 0.3% Tm^3+^ and 4% Yb^3+^, which would indicate this sample as the most promising one in further studies. The optical measurement data including decay times were used to propose the most likely energy transfer diagram between ytterbium and thulium ions. 

The experimental results indicate that these new up-converting nanoparticles are promising materials for biological testing and applications; although, further investigation is mandatory to optimize the properties of the system. Compared to the commonly used nanoparticles like fluorides, bioactive glasses have many advantages, such as non-toxicity, biocompatibility, the possibility of surface functionalization, and more environment-friendly synthesis methods [25,26,42]. The luminescent ions in the amorphous glass are weaker coordinated, so usually, the emission from these ions is less intense and has a lower efficiency compared to fluorides and thus, the way these two materials might be used is different. In fluorides, high emission efficiency is helpful for biomarkers applications. In the case of glasses, that are designed mainly as bioactive materials in regenerative medicine or drug delivery, the UC luminescence can be helpful in biosensing and controlling the compositional and structural changes of the glass.

## Figures and Tables

**Table 1:** Samples names, nominal lanthanides concentration (mol%), and chemical composition (wt.%) of SiO_2_–CaO powders calculated on the basis of a quantitative EDS analysis.

Sample Name	Lanthanides Concentration (mol%)		Chemical Composition (wt.%) ^(a)^			
	Tm^3+^	Yb^3+^	SiO_2_	CaO	Tm_2_O_3_^(b)^	Yb_2_O_3_
0Tm0Yb	0	0	86.8	13.2	-	-
0.15Tm0Yb	0.15	0	86.4	13.6	-	-
0.15Tm1Yb	0.15	1	86.5	9.6	-	3.9
0.15Tm2Yb	0.15	2	76.5	16.3	-	7.2
0.15Tm3Yb	0.15	3	73.4	15.8	-	10.8
0.15Tm4Yb	0.15	4	70,7	16.9	-	12.4
0.3Tm4Yb	0.3	4	69.4	18.0	-	12.6
0.5Tm4Yb	0.5	4	69.5	16.7	1.2	12.6

^(a)^ The relative errors of EDS method are less than 2% and 4% for main (above 20%) and major (5–20%) elements, respectively. ^(b)^ The concentration of the Tm element was below the detection limit.

**Table 2:** The 1/e luminescence decay times of the ^1^G_4_ and ^3^H_4_ manifolds of Tm^3+^ of the silica–calcia powders doped with different Tm^3+^ and Yb^3+^ concentrations.

Tm^3+^ (mol%)	Yb^3+^ (mol%)	Yb^3+^:Tm^3+^ Concentration Ratio	1/e Decay Time (µs)		
			^1^G_4_–^3^H_6_ Transition (@475 nm)	^1^G_4_–^3^F_4_ Transition (@651 nm)	^3^H_4_–^3^H_6_, ^1^G_4_–^3^H_5_ Transitions (@805 nm)
0.15	1	7	13 ± 7	15 ± 8	14 ± 2
0.15	2	13	63 ± 7	27 ± 14	36 ± 5
0.15	3	20	41 ± 14	23 ± 6	31 ± 2
0.15	4	27	63 ± 14	34 ± 4	31 ± 2
0.3	4	13	104 ± 28	109 ± 9	39 ± 5
0.5	4	8	19 ± 2	15 ± 4	13 ± 1

**Table 3:** Values of τ_1_, τ_2_, and A of the double exponential function fitting of the time-resolved UC photoluminescence at 805 nm emission band of Tm^3+^ of the 0.3Tm4Yb powder. The values of ^1^G_4_ decay times are also listed for comparison.

Double Exponential Function Fitting of 805 nm Decay Curve (^1^G_4_ + ^3^H_4_)					^1^G_4_ Decay Time (µs)	
Long decay component		Short decay component		R-square	Derived from ^1^G_4_–^3^H_6_ Transition (@475 nm)	Derived from ^1^G_4_–^3^F_4_ Transition (@651 nm)
A	τ_1_ (µs)	1-A	τ_2_ (µs)			
0.55 ± 0.01	88 ± 2	0.45 ± 0.01	9 ± 1	0.998	104 ± 28	109 ± 9

**Table 4:** The summary of N numbers of different UC photoluminescence at 366 nm, 475 nm, 651 nm, and 805 nm for different concentrations of Tm^3+^ and Yb^3+^ in silica–calcia powders.

Tm^3+^ (mol%)	Yb^3+^ (mol%)	N Number			
		366 nm	475 nm	651 nm	805 nm
0.15	1	(0.32)	2.07	1.56	1.26
0.15	2	1.62	1.95	1.82	1.08
0.15	3	1.67	1.83	1.76	1.04
0.15	4	1.65	1.57	1.51	1.11
0.3	4	2.12	1.50	1.43	0.70
0.5	4	1.73	1.41	1.43	0.75

## Data Availability

Data sharing is not applicable to this article.
